# Supplementary material for: Challenges in explaining deep learning models for data with biological variation
Source: PLoS One. 2025 Oct 9;20(10):e0333965. doi: 10.1371/journal.pone.0333965 (PMC12510501; doi:10.1371/journal.pone.0333965)
Supplement: S1 Appendix — Training hyperparameters, figures of explanations, and detailed results. (PDF) [file pone.0333965.s001.pdf]

Appendix

A Training hyperparameters

To get the best models, we conducted a Bayesian hyperparameter search over the values described in Supplementary Table 2. We then chose the models based on the loss on the validation set. The best hyperparameters are shown in Supplementary Table 1.

Supplementary Table 1. Final hypermarameters.

	Pink fusarium	Skinned
batch size	64	64
# of epochs	100	100
LR scheduler	reduce on plateau	cosine with restarts
LR at start	0.02082	0.00383
weight decay	0.00184	0.78410

Hyperparameters used for training the best models for each disease/damage type.

Supplementary Table 2. Hyperparameter search.

Hyperparameter	Values
# of epochs	max 100 with early stopping
LR scheduler type	{linear, constant, cosine with restarts, reduce LR on plateau }
LR at start	[-12, -2.3] (log-uniform)
weight decay	[-12, 2.3] (log-uniform)

Values of training hyperparameters used in a Bayesian search.

B Figures of explanations

B.1 Pink Fusarium

Supplementary Fig 1 shows examples of grains with pink fusarium disease, ground truth annotations and explanations from all methods used in this paper. EpsilonPlusFlat (LRP-ε-rule for dense layers, LRP-α,β ($\alpha = 1, \beta = 0$), also called ZPlus rule, for convolutional layers, and the flat rule for the first linear layer), EpsilonGammaBox (LRP-ε-rule for dense layers, the LRP-γ-rule ($\gamma = 0.25$) for convolutional layers, and the LRP-Z^B-rule (or box-rule) for the first layer) and EpsilonAlpha2Beta1Flat (LRP-ε-rule for dense layers, LRP-α,β ($\alpha = 2, \beta = 1$) for convolutional layers and the flat rule for the first linear layer) [11].

B.2 Skinned

Supplementary Fig 2 shows examples of grains with a damage "skinned", ground truth annotations and explanations from all methods used in this paper.

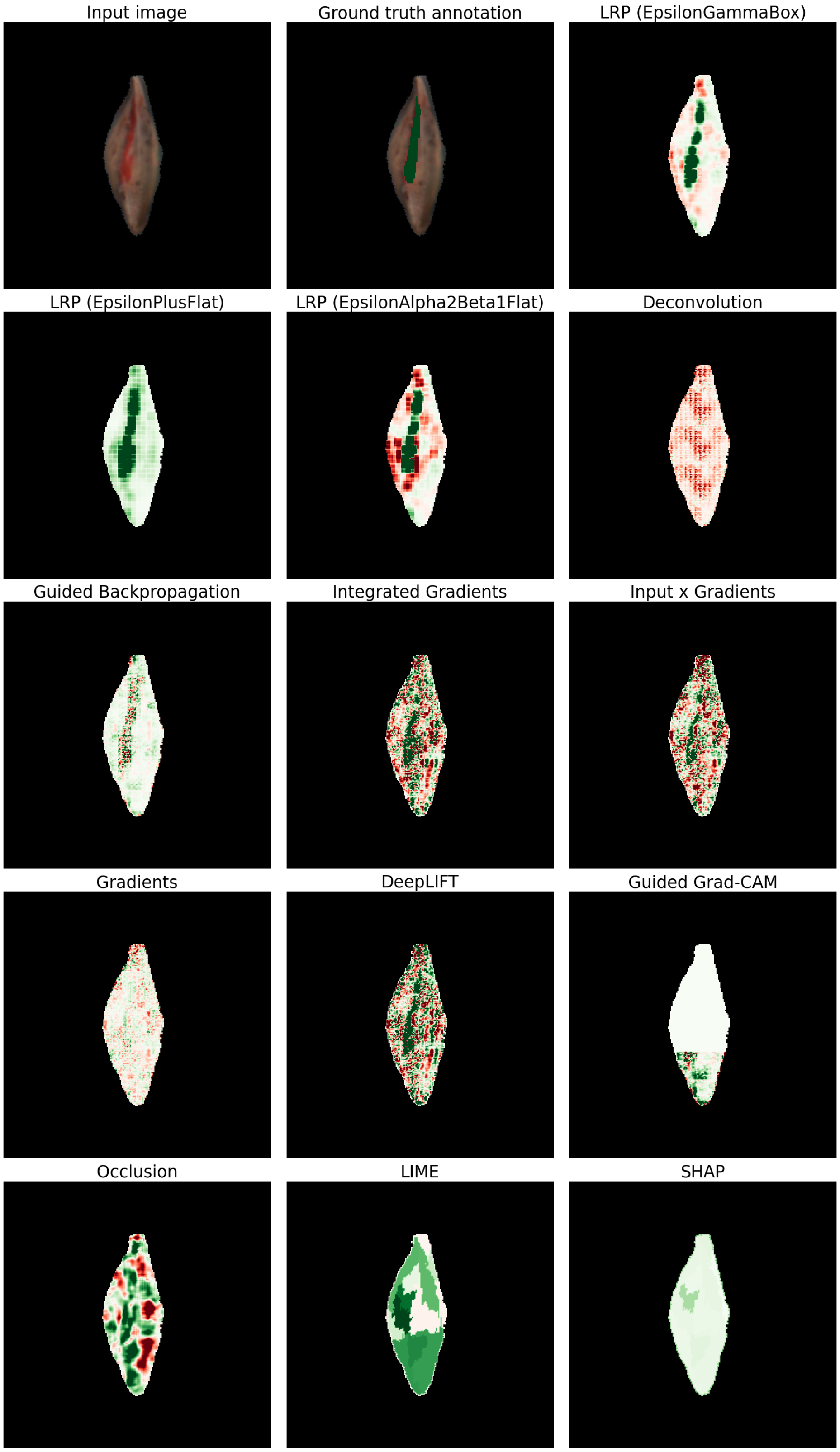
Input image
Ground truth annotation
LRP (EpsilonGammaBox)
LRP (EpsilonPlusFlat)
LRP (EpsilonAlpha2Beta1Flat)
Deconvolution
Guided Backpropagation
Integrated Gradients
Input x Gradients
Gradients
DeepLIFT
Guided Grad-CAM
Occlusion
LIME
SHAP

(a)

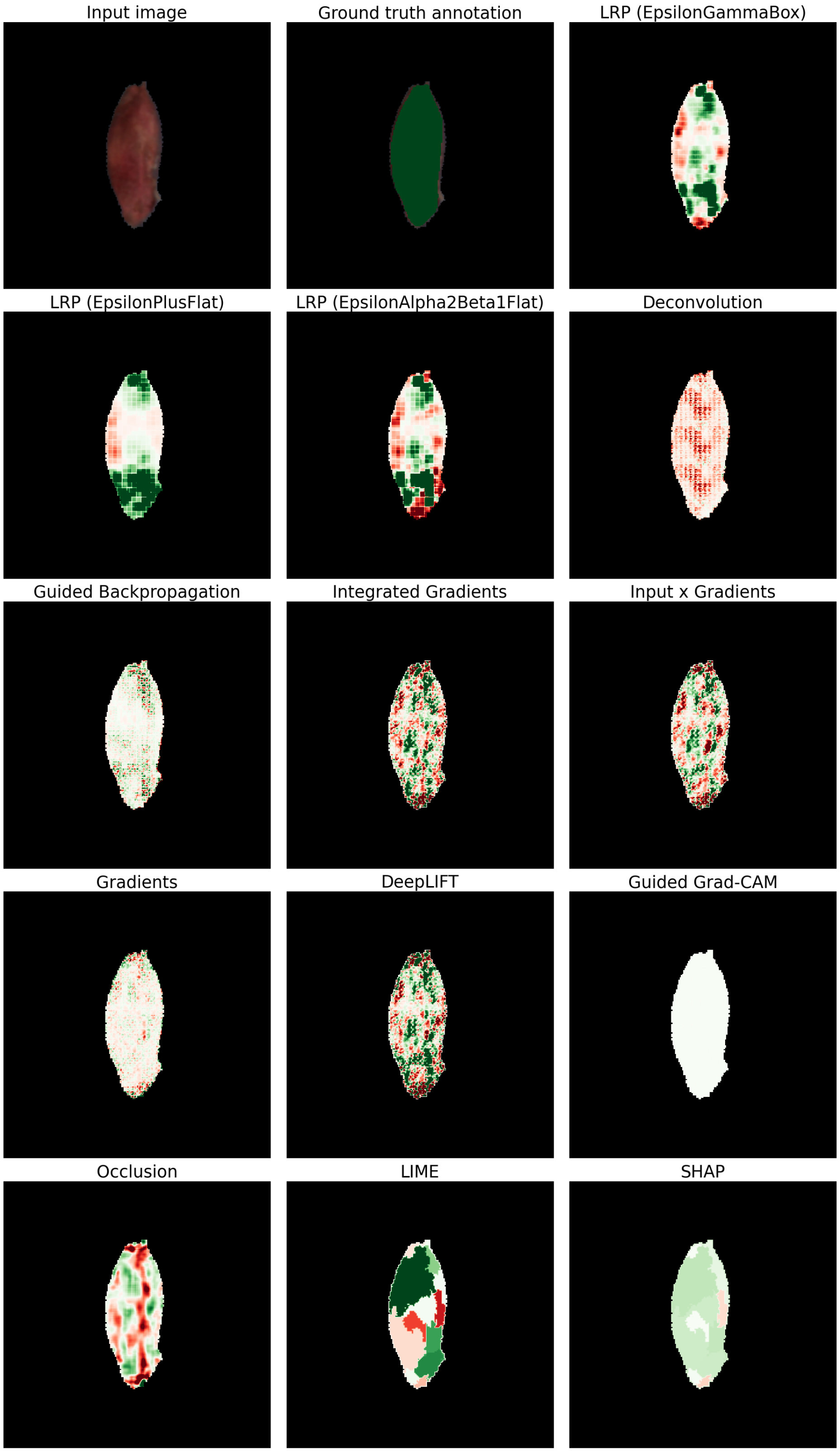
Input image
Ground truth annotation
LRP (EpsilonGammaBox)
LRP (EpsilonPlusFlat)
LRP (EpsilonAlpha2Beta1Flat)
Deconvolution
Guided Backpropagation
Integrated Gradients
Input x Gradients
Gradients
DeepLIFT
Guided Grad-CAM
Occlusion
LIME
SHAP

(b)

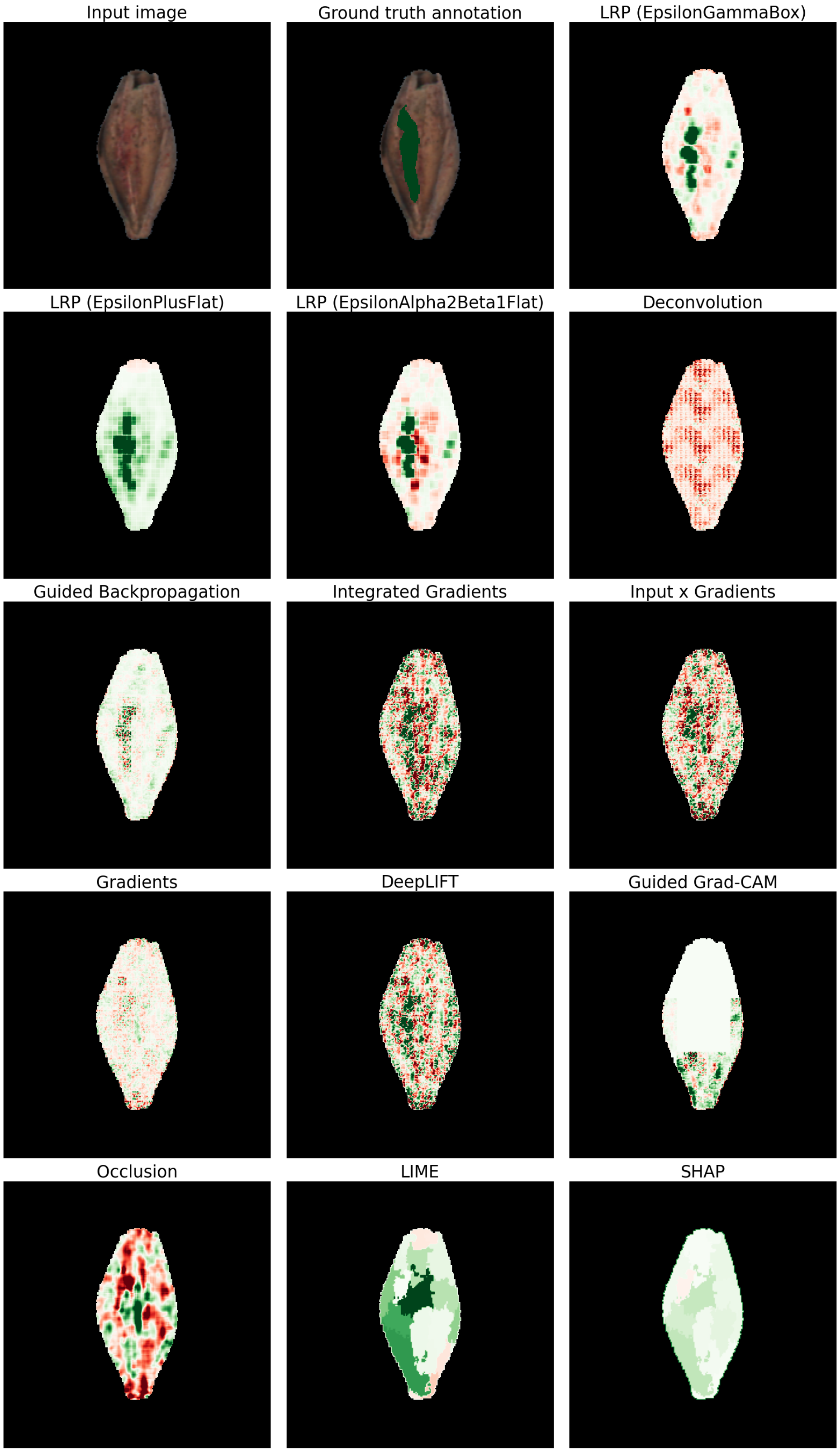

Input image
Ground truth annotation
LRP (EpsilonGammaBox)
LRP (EpsilonPlusFlat)
LRP (EpsilonAlpha2Beta1Flat)
Deconvolution
Guided Backpropagation
Integrated Gradients
Input x Gradients
Gradients
DeepLIFT
Guided Grad-CAM
Occlusion
LIME
SHAP

(c)

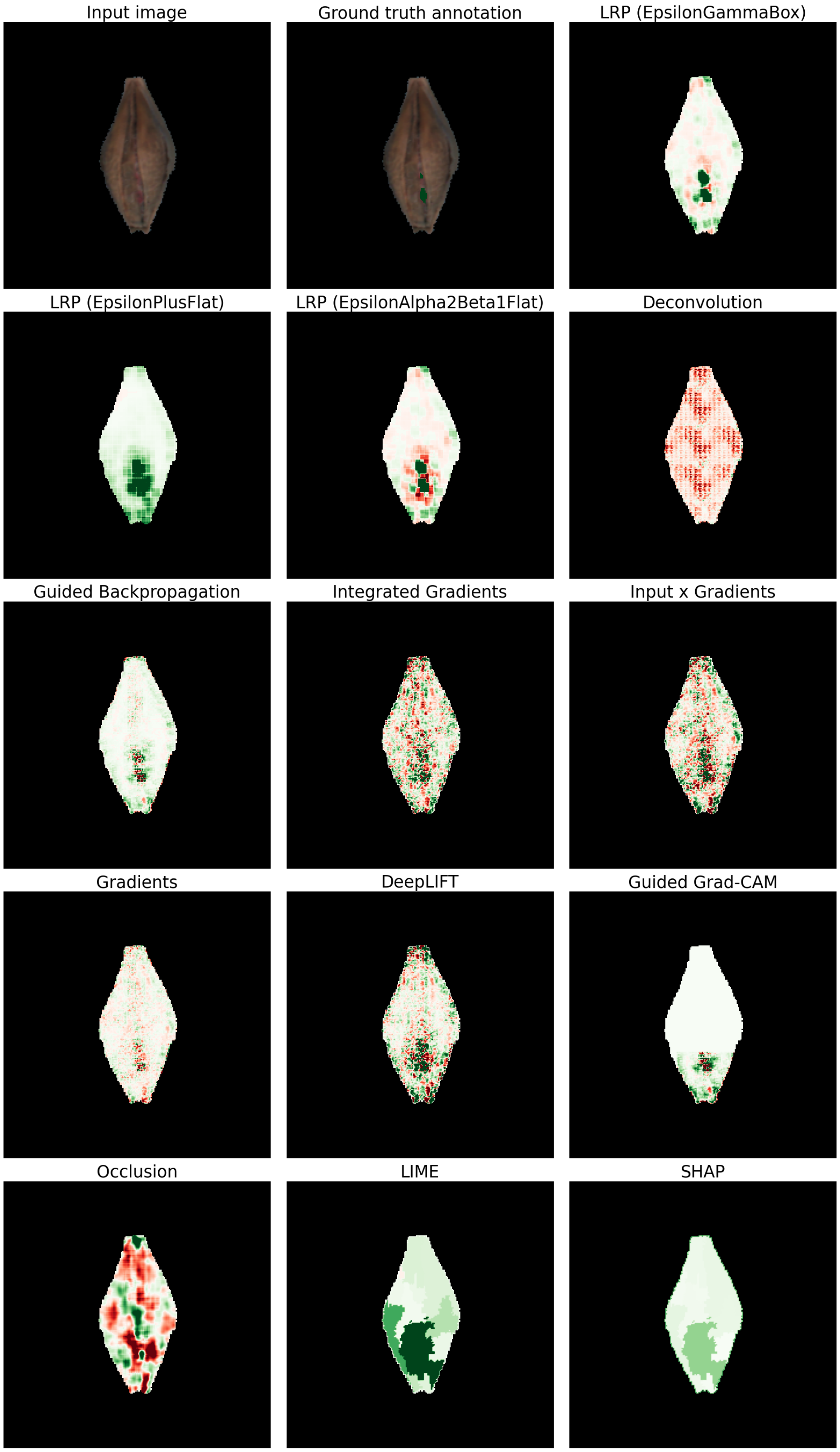
Input image
Ground truth annotation
LRP (EpsilonGammaBox)
LRP (EpsilonPlusFlat)
LRP (EpsilonAlpha2Beta1Flat)
Deconvolution
Guided Backpropagation
Integrated Gradients
Input x Gradients
Gradients
DeepLIFT
Guided Grad-CAM
Occlusion
LIME
SHAP

(d)

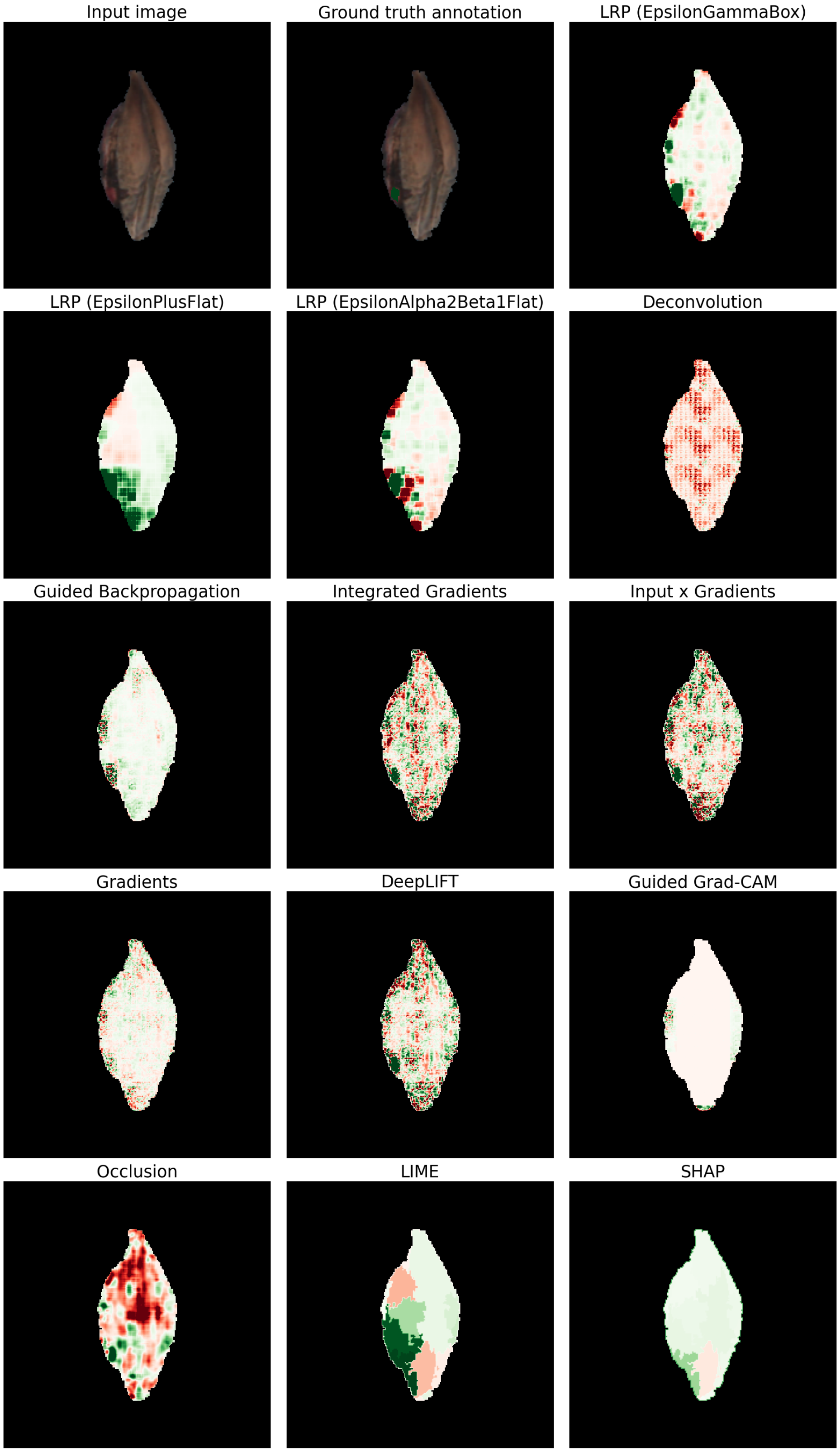

(e)

Supplementary Fig 1. Examples of visualization of explanations for five kernels with the disease pink fusarium. For each kernel, we show the input image, human (ground truth) annotation and explanations produced by all explainability methods used in the paper, namely: three variants of Layer-wise Relevance Propagation (LRP) [1, 2], Deconvolution [3], Guided Backpropagation [4], Integrated Gradients [5], Input x Gradients [6], Gradients [6], Deep Learning Important FeaTures (DeepLIFT) [7], Guided Gradient-weighted Class Activation Mapping (Guided Grad-CAM) [8], Occlusion , Local Interpretable Model-Agnostic Explanations (LIME) [9], and SHapley Additive exPlanations (SHAP) [10]. Green colour means positive attribution, white is neutral, and red is negative attribution.

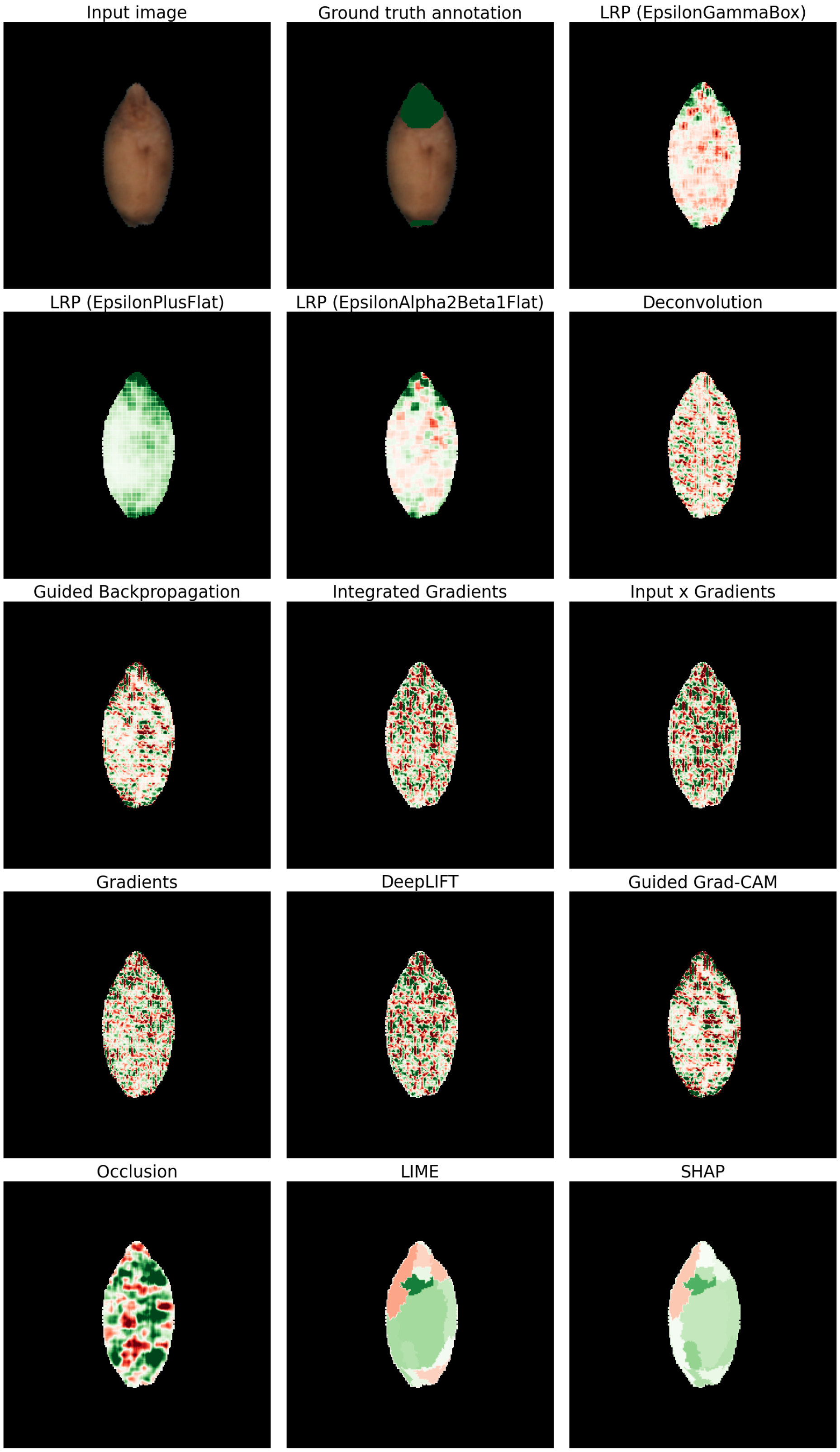
Input image
Ground truth annotation
LRP (EpsilonGammaBox)
LRP (EpsilonPlusFlat)
LRP (EpsilonAlpha2Beta1Flat)
Deconvolution
Guided Backpropagation
Integrated Gradients
Input x Gradients
Gradients
DeepLIFT
Guided Grad-CAM
Occlusion
LIME
SHAP

(a)

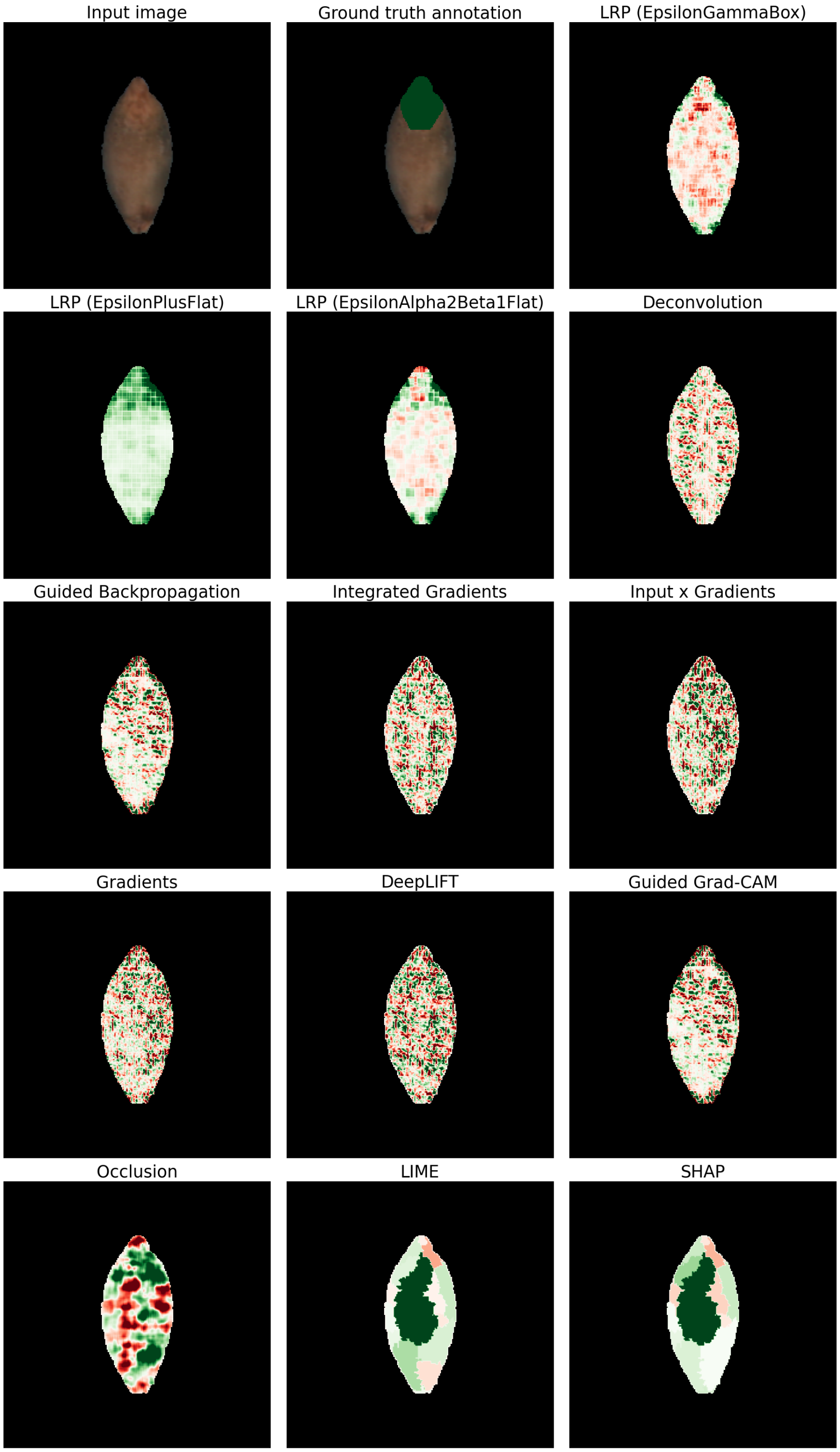
Input image
Ground truth annotation
LRP (EpsilonGammaBox)
LRP (EpsilonPlusFlat)
LRP (EpsilonAlpha2Beta1Flat)
Deconvolution
Guided Backpropagation
Integrated Gradients
Input x Gradients
Gradients
DeepLIFT
Guided Grad-CAM
Occlusion
LIME
SHAP

(b)

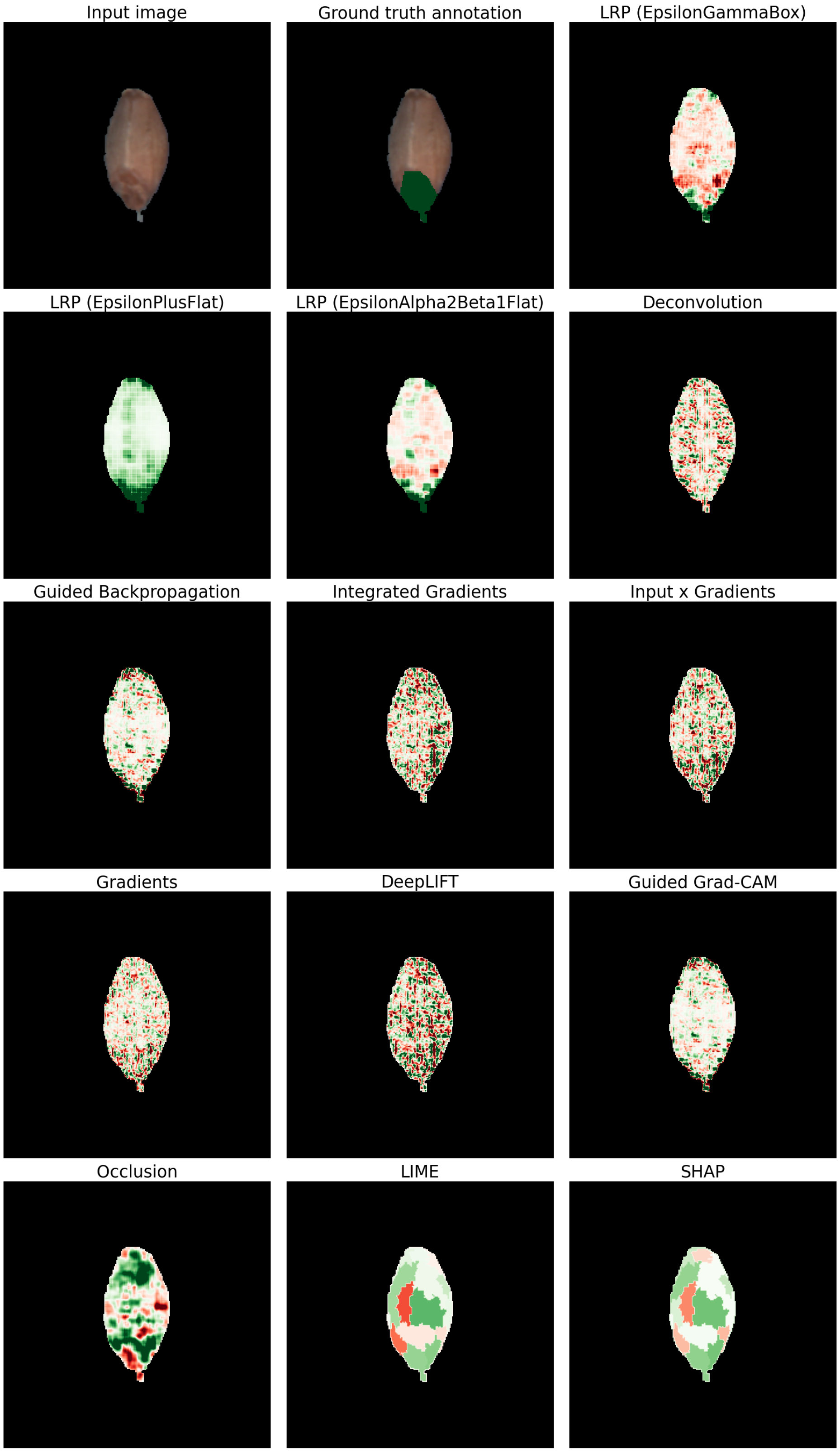
Input image
Ground truth annotation
LRP (EpsilonGammaBox)
LRP (EpsilonPlusFlat)
LRP (EpsilonAlpha2Beta1Flat)
Deconvolution
Guided Backpropagation
Integrated Gradients
Input x Gradients
Gradients
DeepLIFT
Guided Grad-CAM
Occlusion
LIME
SHAP

(c)

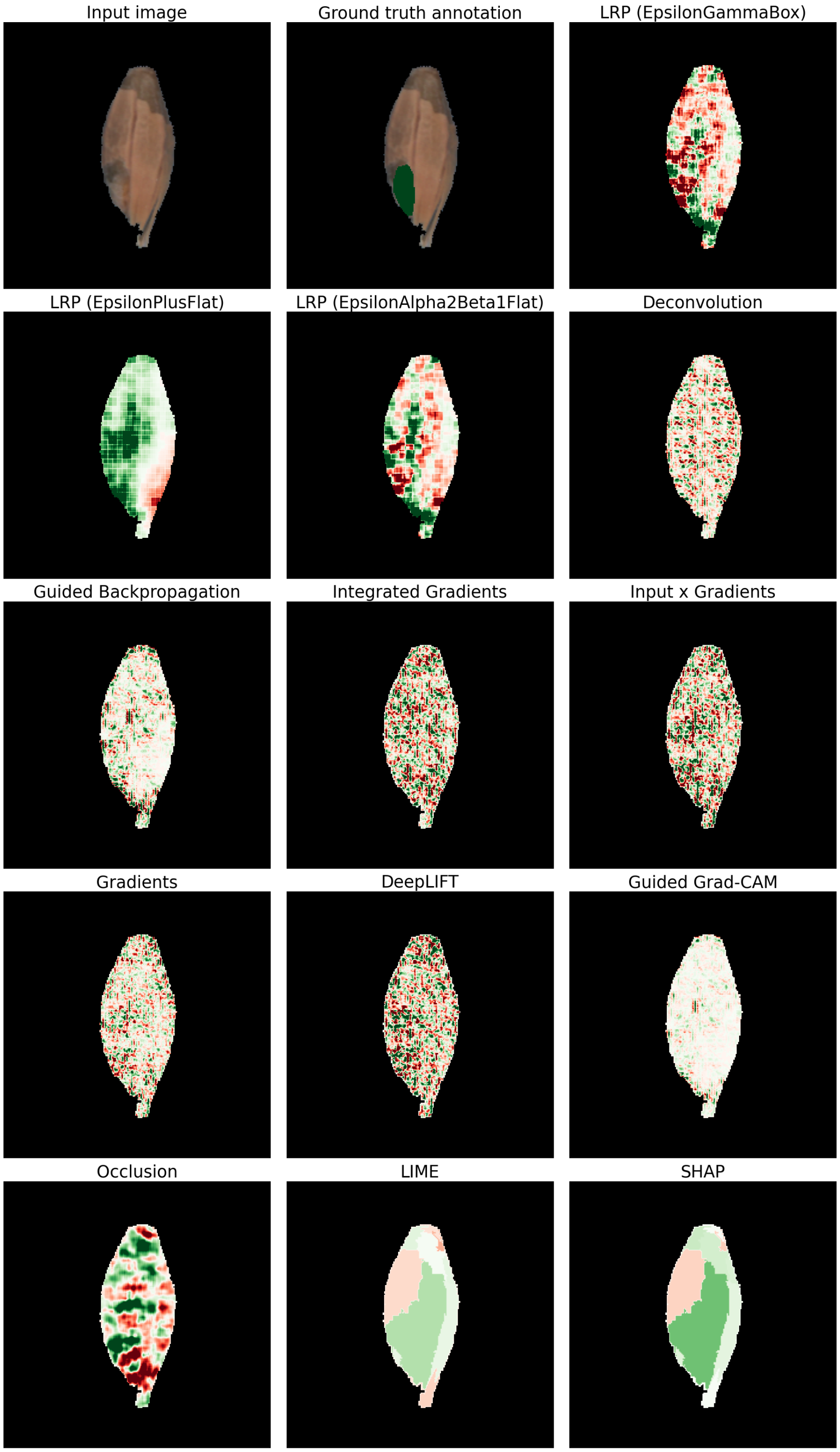
Input image
Ground truth annotation
LRP (EpsilonGammaBox)
LRP (EpsilonPlusFlat)
LRP (EpsilonAlpha2Beta1Flat)
Deconvolution
Guided Backpropagation
Integrated Gradients
Input x Gradients
Gradients
DeepLIFT
Guided Grad-CAM
Occlusion
LIME
SHAP

(d)

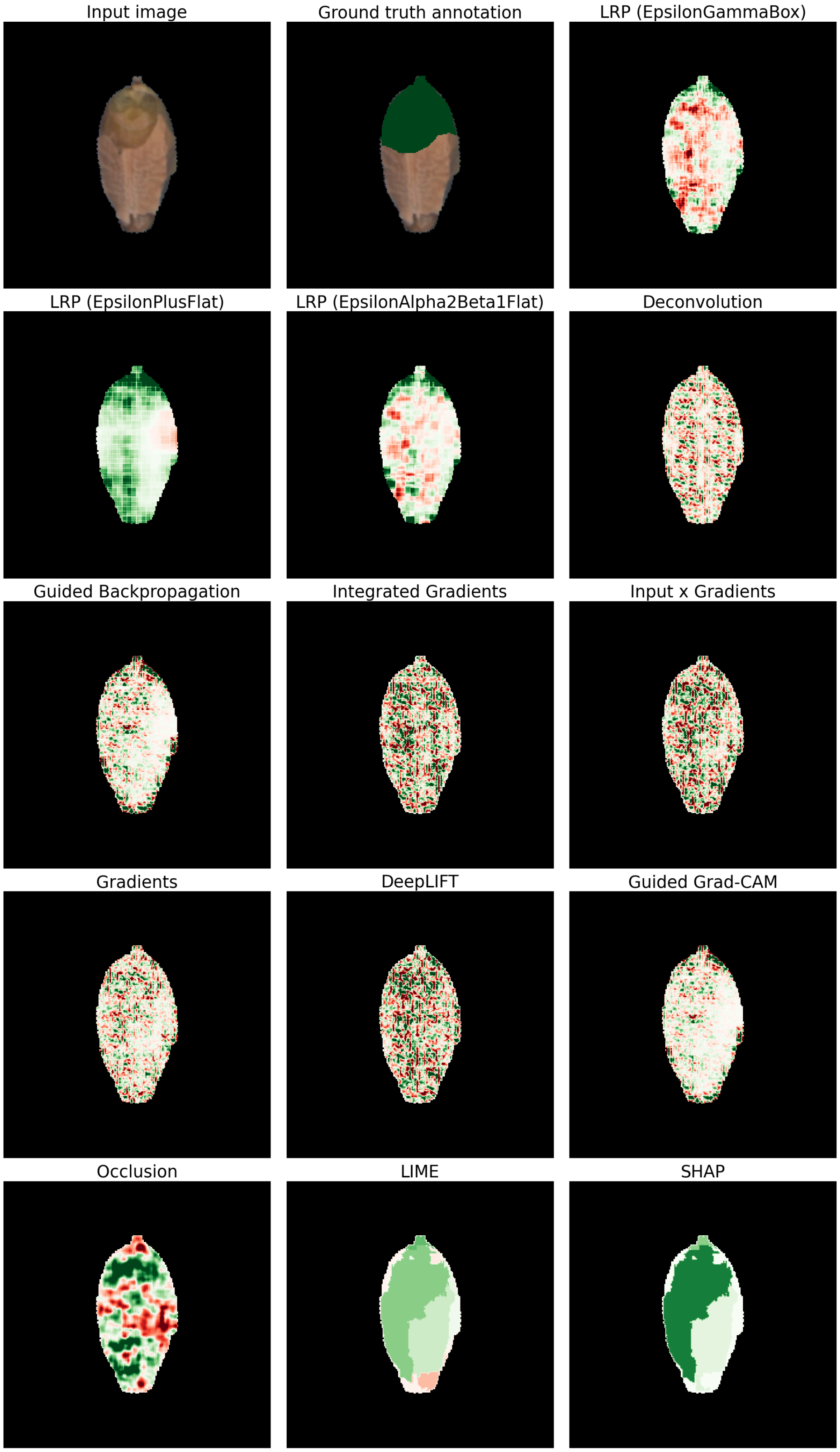

(e)

Supplementary Fig 2. Examples of visualization of explanations for five kernels with the damage "skinned". For each kernel, we show the input image, human (ground truth) annotation and explanations produced by all explainability methods used in the paper, namely: three variants of Layer-wise Relevance Propagation (LRP) [1, 2], Deconvolution [3], Guided Backpropagation [4], Integrated Gradients [5], Input x Gradients [6], Gradients [6], Deep Learning Important FeaTures (DeepLIFT) [7], Guided Gradient-weighted Class Activation Mapping (Guided Grad-CAM) [8], Occlusion , Local Interpretable Model-Agnostic Explanations (LIME) [9], and SHapley Additive exPlanations (SHAP) [10]. Green colour means positive attribution, white is neutral, and red is negative attribution.

C Detailed results

We show the detailed results of our experiments for each metric, hyperparamater choice and explainability method, namely three variants of Layer-wise Relevance Propagation (LRP) [1, 2], Deconvolution [3], Guided Backpropagation [4], Integrated Gradients [5], Input x Gradients [6], Gradients [6], Deep Learning Important FeaTures (DeepLIFT) [7], Guided Gradient-weighted Class Activation Mapping (Guided Grad-CAM) [8], Occlusion , Local Interpretable Model-Agnostic Explanations (LIME) [9], and SHapley Additive exPlanations (SHAP) [10].

C.1 Pink Fusarium

The detailed results can be found in the following tables: robustness to data augmentation in Supplementary Table 3, pixel flipping in Supplementary Table 4, IROF in Supplementary Table 5, sensitivity and complexity in Supplementary Table 6, ROC-AUC in Supplementary Table 7, and Relevance Mass Accuracy in Supplementary Table 8.

Supplementary Table 3. Pink fusarium: Robustness ↑ to data augmentation as described in Section 3.4 .

	Brightness [-10, 10]	Hue [-5, 5]	Saturation [-55, 55]	Rotate [-40, 40]	Scale [0.8, 1.2]	Translate [-0.08, 0.08]
Gradients	0.863±0.007	1.002±0.007	0.894±0.006	0.364±0.007	0.537±0.008	0.415±0.009
Input x Gradients	0.909±0.011	0.907±0.010	0.783±0.010	0.408±0.013	0.623±0.013	0.398±0.012
Integrated Gradients	0.973±0.011	0.978±0.010	0.903±0.010	0.444±0.016	0.692±0.013	0.418±0.015
Deconvolution	**1.059**±0.007	**1.128**±0.008	**1.045**±0.007	0.420±0.006	0.593±0.007	0.465±0.008
Guided Backprop	0.947±0.009	1.069±0.007	0.979±0.006	0.630±0.013	0.710±0.012	**0.660**±0.012
LRP EpsilonPlusFlat	0.914±0.006	0.912±0.007	0.891±0.005	**0.704**±0.009	**0.819**±0.006	**0.653**±0.012
LRP EpsilonGammaBox	0.872±0.006	0.893±0.006	0.870±0.005	0.550±0.011	0.708±0.008	0.536±0.010
LRP Epsilon $\alpha 2 \beta 1$ Flat	0.877±0.006	0.896±0.006	0.862±0.005	0.583±0.009	0.743±0.007	0.552±0.011
DeepLIFT	0.961±0.011	1.000±0.010	0.902±0.009	0.515±0.013	0.710±0.014	0.499±0.012
SHAP	0.863±0.007	1.002±0.007	0.894±0.006	0.364±0.007	0.537±0.008	0.415±0.009
LIME	0.863±0.007	1.002±0.007	0.894±0.006	0.364±0.007	0.537±0.008	0.415±0.009
Occlusion	0.864±0.014	0.970 ± 0.007	-	-	-	-

We change brightness, hue, saturation, rotation, scaling, and translation with values within the corresponding intervals and compare the explanations to the explanations of the original images. Uncertainties are the standard errors of the mean (389 correctly classified images). The intervals for each augmentation method were chosen, so that the probability of the target class drops by at least 10% at one of the endpoints. The first three methods are considered invariant and the latter ones are equivariant. Values below 1 mean that the explanations are less robust than the probabilities of the classifier, higher values signalize higher robustness of explanations when compared to the robustness of the model itself.

Supplementary Table 4. **Pink fusarium: pixel-flipping ↑ for each explainability method and channel pooling type.**

	mean pooling	max pooling	max abs pooling	ℓ_2-norm pooling
Gradients	0.697 ± 0.020	0.390 ± 0.017	0.304 ± 0.016	0.307 ± 0.016
Input x Gradients	**0.738** ± 0.020	0.393 ± 0.016	0.349 ± 0.017	0.349 ± 0.017
Integrated Gradients	**0.741** ± 0.019	0.412 ± 0.017	0.357 ± 0.017	0.357 ± 0.017
Guided Backprop	0.100 ± 0.010	0.388 ± 0.017	**0.431** ± 0.019	**0.432** ± 0.019
Deconvolution	0.028 ± 0.006	0.063 ± 0.009	0.256 ± 0.016	0.260 ± 0.016
LRP: EpsilonPlusFlat	0.446 ± 0.019	0.446 ± 0.019	0.407 ± 0.018	0.409 ± 0.018
LRP: EpsilonGammaBox	0.492 ± 0.021	**0.506** ± 0.021	0.385 ± 0.017	0.387 ± 0.017
LRP: EpsilonAlpha2Beta1Flat	0.391 ± 0.021	0.392 ± 0.021	0.391 ± 0.017	0.391 ± 0.017
DeepLIFT	0.697 ± 0.020	0.425 ± 0.017	0.356 ± 0.017	0.356 ± 0.017
Guided Grad-CAM	0.079 ± 0.010	0.148 ± 0.013	0.150 ± 0.014	0.151 ± 0.014
LIME	0.406 ± 0.018		0.336 ± 0.017	
SHAP	0.091 ± 0.010		0.036 ± 0.008	
Occlusion	0.490 ± 0.021		0.365 ± 0.016	
mean	**0.727** ± 0.019	**0.506** ± 0.017	0.393 ± 0.018	0.393 ± 0.018
ground-truth	0.180 ± 0.016			

In the columns, four variants of channel pooling (mean, max, max abs, and ℓ_2-norm) are shown. Uncertainties are the standard error of the mean (389 images).

Supplementary Table 5. **PinkFusarium: Iterative Removal Of Features (IROF) ↑ for each explainability method and channel pooling type**.

	mean pooling	max pooling	max abs pooling	ℓ_2-norm pooling
Gradients	0.214 ± 0.012	0.224 ± 0.013	0.218 ± 0.013	0.219 ± 0.013
Input x Gradients	0.258 ± 0.014	0.216 ± 0.012	0.231 ± 0.013	0.233 ± 0.013
Integrated Gradients	0.248 ± 0.013	0.225 ± 0.012	0.239 ± 0.013	0.240 ± 0.013
Guided Backprop	0.135 ± 0.007	0.257 ± 0.013	0.274 ± 0.014	0.274 ± 0.014
Deconvolution	0.197 ± 0.014	0.163 ± 0.009	0.225 ± 0.012	0.223 ± 0.012
LRP: EpsilonPlusFlat	0.282 ± 0.015	0.282 ± 0.015	0.282 ± 0.015	0.280 ± 0.014
LRP: EpsilonGammaBox	0.273 ± 0.014	0.276 ± 0.013	0.262 ± 0.014	0.260 ± 0.014
LRP: EpsilonAlpha2Beta1Flat	0.253 ± 0.013	0.254 ± 0.013	0.268 ± 0.014	0.268 ± 0.014
DeepLIFT	0.252 ± 0.013	0.223 ± 0.012	0.226 ± 0.012	0.227 ± 0.013
Guided Grad-CAM	0.143 ± 0.009	0.156 ± 0.010	0.152 ± 0.011	0.152 ± 0.011
LIME	0.376 ± 0.017		0.304 ± 0.016	
SHAP	**0.475** ± 0.019		**0.369** ± 0.021	
Occlusion	0.232 ± 0.013		0.240 ± 0.014	
mean	0.361 ± 0.015	0.367 ± 0.016	0.296 ± 0.016	0.297 ± 0.016

In the columns, four variants of channel pooling (mean, max, max abs, and ℓ_2-norm) are shown. Uncertainties are the standard error of the mean (389 images).

Supplementary Table 6. PinkFusarium: average sensitivity ↓ and complexity ↓ for each explainability method.

	Average Sensitivity ↓	Complexity ↓
Gradients	4.174 ± 0.118	8.822 ± 0.022
Input x Gradients	2.373 ± 0.068	8.835 ± 0.022
Integrated Gradients	2.730 ± 0.090	8.868 ± 0.022
Deconvolution	2.237 ± 0.034	8.796 ± 0.023
Guided Backpropagation	12.194 ± 0.691	8.337 ± 0.023
LRP: EpsilonPlusFlat	1.443 ± 0.026	8.766 ± 0.027
LRP: EpsilonGammaBox	**1.347** ± 0.020	8.554 ± 0.027
LRP: EpsilonAlpha2Beta1Flat	**1.330** ± 0.020	8.498 ± 0.029
DeepLIFT	4.312 ± 0.134	8.747 ± 0.022
Guided Grad-CAM	16.595 ± 14.708	**7.006** ± 0.434
Occlusion	2.611 ± 0.072	9.126 ± 0.023

Uncertainties are the standard errors of the mean (389 images).

Supplementary Table 7. Pink fusarium: ROC-AUC ↑ for each explainability method and channel pooling type.

	mean pooling	max pooling	max abs pooling	ℓ_2-norm pooling
Gradients	0.731 ± 0.001	0.958 ± 0.001	0.955 ± 0.002	0.956 ± 0.002
Input x Gradients	0.756 ± 0.002	0.963 ± 0.001	0.973 ± 0.001	0.973 ± 0.001
Integrated Gradients	0.765 ± 0.003	0.963 ± 0.001	0.975 ± 0.001	0.975 ± 0.001
Guided Backpropagation	0.787 ± 0.001	0.973 ± 0.001	0.979 ± 0.001	0.979 ± 0.001
Deconvolution	0.637 ± 0.001	0.816 ± 0.002	0.858 ± 0.002	0.858 ± 0.002
LRP: EpsilonPlusFlat	**0.965**. ± 0.005	**0.965** ± 0.005	**0.982** ± 0.001	**0.982** ± 0.001
LRP: EpsilonGammaBox	0.848 ± 0.006	0.877 ± 0.005	**0.983** ± 0.001	**0.983** ± 0.001
LRP: EpsilonAlpha2Beta1Flat	0.825 ± 0.006	0.825 ± 0.006	0.980 ± 0.001	0.980 ± 0.001
DeepLIFT	0.781 ± 0.002	0.966 ± 0.001	0.976 ± 0.001	0.976 ± 0.001
GuidedGradCam	0.715 ± 0.011	0.751 ± 0.013	0.731 ± 0.014	0.731 ± 0.014
LIME	0.901 ± 0.007		0.963 ± 0.003	
SHAP	0.592 ± 0.012		0.592 ± 0.012	
Occlusion	0.726 ± 0.006		0.966 ± 0.001	
mean	0.820 ± 0.004	**0.964** ± 0.002	**0.983** ± 0.001	**0.983** ± 0.001

In the columns, four variants of channel pooling (mean, max, max abs, and ℓ_2-norm) are shown. Uncertainties are the standard errors of the mean (173 images).

Supplementary Table 8. **Pink fusarium: Relevance Mass Accuracy ↑ for each explainability method.**

	max abs pooling	ℓ_2-norm pooling
Gradients	0.125 ± 0.005	0.128 ± 0.005
Input x Gradients	0.373 ± 0.015	0.380 ± 0.015
Integrated Gradients	0.402 ± 0.016	0.409 ± 0.016
Deconvolution	0.050 ± 0.003	0.050 ± 0.003
Guided Backpropagation	0.405 ± 0.010	0.412 ± 0.011
LRP: EpsilonPlusFlat	**0.485** ± 0.014	**0.485** ± 0.014
LRP: EpsilonGammaBox	0.469 ± 0.012	0.458 ± 0.012
LRP: EpsilonAlpha2Beta1Flat	0.551 ± 0.013	0.551 ± 0.013
DeepLIFT	0.428 ± 0.015	0.437 ± 0.015
Guided Grad-CAM	0.143 ± 0.143	0.146 ± 0.146
mean	0.350 ± 0.017	0.350 ± 0.017

In the columns, two variants of channel pooling (max abs and ℓ_2-norm) are shown. Uncertainties are the standard error of the mean (173 images). Only max abs and ℓ_2-norm pooling are included since this method expects non-negative explanations.

C.2 Skinned

The detailed results can be found in the following tables: robustness to data augmentation in Supplementary Table 9, pixel flipping in Supplementary Table 10, IROF in Supplementary Table 11, sensitivity and complexity in Supplementary Table 12, ROC-AUC in Supplementary Table 13, and Relevance Mass Accuracy in Supplementary Table 14.

Supplementary Table 9. Skinned: Robustness ↑ to data augmentationas described in Section 3.4.

	Brightness [-20, 20]	Hue [-15, 15]	Saturation [-50, 50]	Rotate [-8, 8]	Scale [0.8, 1.2]	Translate [-0.04, 0.04]
Gradients	0.838±0.007	0.883±0.006	0.871±0.006	0.317±0.003	0.249±0.004	0.378±0.005
Input x Gradients	0.863±0.011	0.807±0.009	0.805±0.008	0.405±0.007	0.345±0.006	0.422±0.008
Integrated Gradients	1.004±0.011	0.931±0.009	0.950±0.009	0.448±0.007	0.405±0.007	0.429±0.007
Deconvolution	**1.050**±0.008	**1.037**±0.007	**1.034**±0.007	0.333±0.003	0.277±0.003	0.481±0.006
Guided Backprop	0.899±0.007	0.935±0.006	0.923±0.006	0.485±0.005	0.445±0.006	0.557±0.007
LRP EpsilonPlusFlat	0.927±0.006	0.953±0.005	0.953±0.005	**0.718**±0.010	**0.701**±0.010	**0.632**±0.012
LRP EpsilonGammaBox	0.889±0.005	0.927±0.004	0.923±0.004	0.471±0.005	0.408±0.005	0.419±0.006
LRP EpsilonAlpha2Beta1Flat	0.910±0.006	0.938±0.005	0.934±0.005	0.587±0.008	0.552±0.009	0.498±0.009
DeepLIFT	0.945±0.011	0.907±0.009	0.915±0.008	0.415±0.006	0.363±0.005	0.373±0.006
SHAP	0.838±0.007	0.883±0.006	0.871±0.006	0.317±0.003	0.249±0.004	0.378±0.005
LIME	0.838±0.007	0.883±0.006	0.871±0.006	0.317±0.003	0.249±0.004	0.378±0.005
Occlusion	0.930±0.011	-	-	-	-	-

We change brightness, hue, saturation, rotation, scaling, and translation with values within the corresponding intervals and compare the explanations to the explanations of the original images. Uncertainties are the standard errors of the mean (383 correctly classified images). The intervals for each augmentation method were chosen, so that the probability of the target class drops by at least 10% at one of the endpoints. The first three methods are considered invariant and the latter ones are equivariant. Values below 1 mean that the explanations are less robust than the probabilities of the classifier, higher values signalize higher robustness of explanations when compared to the robustness of the model itself.

Supplementary Table 10. Skinned: pixel-flipping ↑ for each explainability method and channel pooling type.

	mean pooling	max pooling	max abs pooling	ℓ_2-norm pooling
Gradients	0.519 ± 0.025	0.504 ± 0.024	0.538 ± 0.017	0.548 ± 0.017
Input x Gradients	0.503 ± 0.025	0.497 ± 0.025	0.503 ± 0.008	0.529 ± 0.008
Integrated Gradients	0.495 ± 0.025	0.482 ± 0.025	0.528 ± 0.008	0.528 ± 0.008
Guided Backpropagation	0.507 ± 0.024	0.488 ± 0.023	0.557 ± 0.018	0.560 ± 0.018
Deconvolution	0.449 ± 0.022	0.433 ± 0.021	0.449 ± 0.021	0.454 ± 0.021
LRP: EpsilonPlusFlat	0.658 ± 0.013	0.658 ± 0.013	**0.605** ± 0.013	**0.606** ± 0.013
LRP: EpsilonGammaBox	0.573 ± 0.022	0.568 ± 0.022	**0.610** ± 0.015	**0.607** ± 0.015
LRP: EpsilonAlpha2Beta1Flat	0.571 ± 0.022	0.571 ± 0.022	0.568 ± 0.021	0.570 ± 0.020
DeepLIFT	0.498 ± 0.025	0.491 ± 0.025	0.522 ± 0.008	0.520 ± 0.008
Guided Grad-CAM	0.484 ± 0.023	0.465 ± 0.023	0.533 ± 0.018	0.535 ± 0.018
LIME	**0.835** ± 0.011		0.563 ± 0.015	
SHAP	0.755 ± 0.015		0.545 ± 0.017	
Occlusion	0.681 ± 0.019		0.522 ± 0.009	
mean	0.497 ± 0.024	0.517 ± 0.023	0.555 ± 0.009	0.554 ± 0.009
ground-truth	0.499 ± 0.018			

In the columns, four variants of channel pooling (mean, max, max abs, and ℓ_2-norm) are shown. Uncertainties are the standard error of the mean (383 images).

Supplementary Table 11. Skinned: Iterative Removal Of Features (IROF) ↑ for each explainability method and channel pooling type.

	mean pooling	max pooling	max abs pooling	ℓ_2-norm pooling
Gradients	0.365 ± 0.011	0.322 ± 0.010	0.314 ± 0.010	0.313 ± 0.010
Input x Gradients	0.377 ± 0.012	0.333 ± 0.011	0.355 ± 0.011	0.352 ± 0.011
Integrated Gradients	0.316 ± 0.011	0.328 ± 0.010	0.382 ± 0.011	0.375 ± 0.011
Guided Backpropagation	0.288 ± 0.010	0.318 ± 0.011	0.354 ± 0.011	0.351 ± 0.011
Deconvolution	0.269 ± 0.010	0.374 ± 0.010	0.399 ± 0.011	0.394 ± 0.011
LRP: EpsilonPlusFlat	0.408 ± 0.011	0.408 ± 0.011	0.385 ± 0.011	0.385 ± 0.011
LRP: EpsilonGammaBox	0.339 ± 0.011	0.345 ± 0.011	0.340 ± 0.011	0.339 ± 0.011
LRP: EpsilonAlpha2Beta1Flat	0.391 ± 0.011	0.391 ± 0.011	0.347 ± 0.011	0.347 ± 0.011
DeepLIFT	0.366 ± 0.012	0.347 ± 0.010	0.354 ± 0.011	0.352 ± 0.011
Guided Grad-CAM	0.278 ± 0.010	0.314 ± 0.011	0.351 ± 0.011	0.351 ± 0.011
LIME	**0.821** ± 0.010		**0.557** ± 0.014	
SHAP	0.805 ± 0.010		**0.556** ± 0.015	
Occlusion	0.367 ± 0.012		0.391 ± 0.013	
mean	0.696 ± 0.010	0.677 ± 0.010	0.495 ± 0.013	0.496 ± 0.013

In the columns, four variants of channel pooling (mean, max, max abs, and ℓ_2-norm) are shown. Uncertainties are the standard error of the mean (383 images).

Supplementary Table 12. Skinned: average sensitivity ↓ and complexity ↓ for each explainability method.

	Average Sensitivity ↓	Complexity ↓
Gradients	2.850 ± 0.035	9.143 ± 0.010
Input x Gradients	1.807 ± 0.019	9.057 ± 0.010
Integrated Gradients	**1.407** ± 0.007	9.104 ± 0.011
Deconvolution	2.170 ± 0.012	9.192 ± 0.011
Guided Backpropagation	4.344 ± 0.081	8.746 ± 0.011
LRP: EpsilonPlusFlat	1.756 ± 0.044	9.075 ± 0.014
LRP: EpsilonGammaBox	2.001 ± 0.049	9.031 ± 0.012
LRP: EpsilonAlpha2Beta1Flat	2.318 ± 0.066	8.902 ± 0.017
DeepLIFT	1.804 ± 0.014	9.116 ± 0.010
Guided Grad-CAM	22.421 ± 7.328	**8.640** ± 0.024

Uncertainties are the standard error of the mean (383 images).

Supplementary Table 13. Skinned: ROC-AUC ↑ for each explainability method and channel pooling type.

	mean pooling	max pooling	max abs pooling	ℓ_2-norm pooling
Gradients	0.707 ± 0.002	0.838 ± 0.005	0.897 ± 0.005	0.897 ± 0.005
Input x Gradients	0.709 ± 0.002	0.842 ± 0.004	0.903 ± 0.005	0.905 ± 0.005
Integrated Gradients	0.709 ± 0.002	0.842 ± 0.004	0.903 ± 0.005	0.903 ± 0.005
Guided Backpropagation	0.707 ± 0.002	0.817 ± 0.003	0.890 ± 0.004	0.890 ± 0.004
Deconvolution	0.685 ± 0.002	0.776 ± 0.003	0.809 ± 0.003	0.811 ± 0.003
LRP: EpsilonPlusFlat	**0.908** ± 0.005	**0.908** ± 0.005	**0.917** ± 0.005	**0.917** ± 0.005
LRP: EpsilonGammaBox	0.702 ± 0.003	0.765 ± 0.003	0.910 ± 0.005	0.910 ± 0.005
LRP: EpsilonAlpha2Beta1Flat	0.768 ± 0.004	0.768 ± 0.004	0.906 ± 0.005	0.906 ± 0.005
DeepLIFT	0.714 ± 0.002	0.839 ± 0.004	0.904 ± 0.005	0.904 ± 0.005
Guided Grad-CAM	0.709 ± 0.003	0.816 ± 0.003	0.889 ± 0.005	0.889 ± 0.005
LIME	0.809 ± 0.008		0.887 ± 0.005	
SHAP	0.738 ± 0.010		0.772 ± 0.011	
Occlusion	0.754 ± 0.005		0.905 ± 0.005	
mean	0.758 ± 0.003	0.856 ± 0.004	0.910 ± 0.005	0.910 ± 0.005

In the columns, four variants of channel pooling (mean, max, max abs, and ℓ_2-norm) are shown. Uncertainties are the standard error of the mean (145 images).

Supplementary Table 14. Skinned: Relvance Mass Accuracy ↑ for each explainability method and channel pooling type.

	max abs pooling	ℓ_2-norm pooling
Gradients	0.140 ± 0.005	0.138 ± 0.005
Input x Gradients	0.350 ± 0.017	0.349 ± 0.017
Integrated Gradients	0.346 ± 0.017	0.345 ± 0.016
Deconvolution	0.055 ± 0.003	0.056 ± 0.003
Guided Backpropagation	0.246 ± 0.007	0.246 ± 0.007
LRP: EpsilonPlusFlat	**0.363** ± 0.010	**0.363** ± 0.010
LRP: EpsilonGammaBox	0.251 ± 0.007	0.249 ± 0.007
LRP: EpsilonAlpha2Beta1Flat	0.336 ± 0.010	0.336 ± 0.010
DeepLIFT	0.348 ± 0.016	0.347 ± 0.016
Guided Grad-CAM	0.258 ± 0.010	0.257 ± 0.009
mean	0.338 ± 0.016	0.338 ± 0.016

In the columns, two variants of channel pooling (max abs and ℓ_2-norm) are shown. Uncertainties are the standard error of the mean (145 images). Only max abs and ℓ_2-norm pooling are included since this method expects non-negative explanations.

References

1. Bach, S. *et al.* On pixel-wise explanations for non-linear classifier decisions by layer-wise relevance propagation. *PloS one* **10**, e0130140 (2015).

2. Kohlbrenner, M. *et al.* Towards best practice in explaining neural network decisions with lrp. In *2020 International Joint Conference on Neural Networks (IJCNN)*, 1–7 (IEEE, 2020).

3. Zeiler, M. D. & Fergus, R. Visualizing and understanding convolutional networks. In *European conference on computer vision*, 818–833 (Springer, 2014).

4. Springenberg, J. T., Dosovitskiy, A., Brox, T. & Riedmiller, M. Striving for simplicity: The all convolutional net. *arXiv preprint arXiv:1412.6806* (2014).

5. Sundararajan, M., Taly, A. & Yan, Q. Axiomatic attribution for deep networks. In *International conference on machine learning*, 3319–3328 (PMLR, 2017).

6. Simonyan, K., Vedaldi, A. & Zisserman, A. Deep inside convolutional networks: Visualising image classification models and saliency maps. *arXiv preprint arXiv:1312.6034* (2013).

7. Shrikumar, A., Greenside, P. & Kundaje, A. Learning important features through propagating activation differences. In *International conference on machine learning*, 3145–3153 (PMLR, 2017).

8. Selvaraju, R. R. *et al.* Grad-cam: Visual explanations from deep networks via gradient-based localization. In *Proceedings of the IEEE international conference on computer vision*, 618–626 (2017).

9. Ribeiro, M. T., Singh, S. & Guestrin, C. "why should I trust you?": Explaining the predictions of any classifier. In *Proceedings of the 22nd ACM SIGKDD International Conference on Knowledge Discovery and Data Mining, San Francisco, CA, USA, August 13-17, 2016*, 1135–1144 (2016).

10. Lundberg, S. M. & Lee, S.-I. A unified approach to interpreting model predictions. In Guyon, I. *et al.* (eds.) *Advances in Neural Information Processing Systems 30*, 4765–4774 (Curran Associates, Inc., 2017).

11. Montavon, G., Binder, A., Lapuschkin, S., Samek, W. & Müller, K.-R. *Layer-Wise Relevance Propagation: An Overview*, 193–209 (2019). JournalAbbreviation: Lecture Notes in Computer Science (including subseries Lecture Notes in Artificial Intelligence and Lecture Notes in Bioinformatics).
